# The assessment of perioperative risk factors of anastomotic leakage after intestinal surgeries; a prospective study

**DOI:** 10.1186/s12893-020-01044-8

**Published:** 2021-01-07

**Authors:** Selmy Awad, Ahmed Ibrahim Abd El-Rahman, Ashraf Abbas, Waleed Althobaiti, Shaker Alfaran, Saleh Alghamdi, Saleh Alharthi, Khaled Alsubaie, Soliman Ghedan, Rayan Alharthi, Majed Asiri, Azzah Alzahrani, Nawal Alotaibi, Ashraf Shoma, Mohamed Samir Abou Sheishaa

**Affiliations:** 1 General Surgery Department, Mansoura University Hospitals, Mansoura, 35516 Egypt; 2 General Surgery Department, King Faisal Medical Complex, Taif, Saudi Arabia

**Keywords:** Anastomosis, Intestinal, Leakage, Predictors

## Abstract

**Background:**

Anastomotic leaks (AL) are among the most serious complications due to the substantial impact on the quality of life and mortality. Inspite of the advance in diagnostic tools such as laboratory tests and radiological adjuncts, only moderate improvement has been recorded in the rate of detected leaks. The purpose of the research was to assess the perioperative risk factors for AL.

**Methods:**

This study was achieved at MUH and MIH/Egypt within the period between January 2016 and January 2019 for the candidates who underwent bowel anastomosis for small intestinal (except duodenal one) and colorectal pathology. The collected data were analyzed using SPSS of V-26.

**Results:**

This study included 315 cases, among them, 27 cases (8.57%) developed AL. The percentage of covering stoma was significantly higher in the non-leakage group vs leakage one (24.3% vs 11.1% respectively). lower albumin, operative timing, perforation, and covering stoma were shown as significant risk factors for leakage, but with multivariate analysis for these factors, the emergency operation, and serum albumin level was the only independent risk factors that revealed the significance consequently (p = 0.043, p = 0.015). The analysis of different predictors of AL on the third day showed that the cut-off point in RR was 29 with 83% sensitivity and 92% specificity in prediction of leakage, the cut-off point in RR was 118 with 74% sensitivity and 87% specificity in prediction of leakage and the cut-off point in CRP was 184.7 with 82% sensitivity and 88% specificity in prediction of AL and all had statistically significant value.

**Conclusions:**

The preoperative serum albumin level and the emergency operations are independent risk factors for anastomotic leakage. Moreover, leakage should be highly suspected in cases with rising respiratory rate, heart rate, and CRP levels.

## Background

Anastomotic leakage (AL) after colorectal surgery is more common than that is of the small intestine. Its incidence ranges from 2 to 24%. This incidence increases with rectal anastomosis more than colonic anastomosis [1, 2].

Many risk factors are highly considered with AL as nature and level of the primary disease, prolonged operative time, absence of proximal diversion increased blood loss and associated systemic disorders like DM, anemia, chronic obstructive pulmonary disease (COPD), malnutrition, hypoalbuminemia, vitamin deficiency, previous irradiation, steroids, male gender, cigarette smoking, advanced age, and poor hydration status in the emergency setting as a consequence of sepsis, obstruction, or both [3]*.*

Multiple scoring systems were created for predicting the risk of AL after intestinal anastomosis. These risk scores include numerous parameters of the previously mentioned risk factors. These scores should help the surgeons to make more safe decisions regarding the performance of anastomosis versus stoma [4]*.*

The risk factors associated with a lower risk of AL are adequate blood supply, assurance of the tension-free anastomosis, adequate matching of the luminal diameters, proper technical placement of sutures, and adequate preoperative antibiotic prophylaxis [5]. AL places a major impact on both physician and patient. Major leaks manifest early and require rapid aggressive management to avoid the development of complications of AL as sepsis and multiorgan failure [6].

Some postoperative signs are associated with AL as fever, increased leucocytic count, increased CRP level, renal failure, pelvic pain, and peritonitis [7].

Efforts of our two-center were directed to estimate the diagnostic accuracy of the already present parameters and predictors that could be used for prediction and early detection of AL and to be applied for all patients with intestinal anastomosis.

## Methods

*Study design and setting of the study population* The research was a prospective observational study. It included the patients at Mansoura University Hospitals (MUH) and Mansoura International Hospital (MIH), Mansoura city, Egypt who underwent small intestinal or colorectal anastomosis for any pathology in the period between January 2016 and January 2019. Out of 315 cases, 27 cases (8.57%) developed AL after the surgery (leakage group) and the other patients had no leakage (no-leakage group).

*Inclusion criteria *all Patients who underwent a bowel anastomosis for any cause either at the index operation or second stage reconstruction were recruited. The Patient with Lost follow up or missing data, duodenal injuries, pregnancy, and age < 18 years were excluded*.*

*Preoperative workup *Detailed History taking and thorough Clinical examination was done for all cases with Anesthetic consultation to assess the physical status according to the ASA score system.

The investigations were done for all cases including;

(A) Laboratory:

- Routine preoperative investigations
- Tumor markers: baseline CEA and CA 19-9 for suspected cancer cases.
- LDH, CRP, and ABG for cases with suspected mesenteric ischemia or perforation.

(B) Radiological:

- Metastatic workup in cases of malignancy.
- Plain X-ray chest, abdominal X-ray, and the abdominal US
- C.T. abdomen and pelvis.

(C) Colonoscopy: Complete colonoscopy up to beyond the caecum was done in almost all elective patients ± biopsy.

*Preoperative preparation* All elective patients received standard mechanical (preoperative liquid diet and enemas) and chemical bowel preparation (oral metronidazole and neomycin) apart from patients with the right-sided colonic lesions.

- The cases with low albumin or hemoglobin levels were optimized preoperatively.
- The day before the operation, the cases got the prophylactic antibiotics and VTE chemoprophylaxis in patients with high risks.

*The main operative techniques* All patients were performed by qualified surgical teams with standard surgical procedures and techniques. In small intestinal lesions, the anastomosis (end–end type) was done in an interrupted fashion in most of our cases. Forty-two cases (including distal colon anastomosis) were done using the stapler technique. The Open approach was the commonly used one. The diversion was performed based on the clinical indications and the patient's general condition.

*Post-operative care* Most cases were transferred to the ward postoperatively apart from patients demanding intensive ICU monitoring.

- CBC was ordered for all patients daily for the 1st PODs and CRP was withdrawn in the 3rd postoperative day (POD).
- Patients were kept NPO and intravenous fluids and serum electrolytes were optimized.
- The patients, with uneventful postoperative courses, were discharged on the 5th-7th PODs.
- AL was suspected in the presence of abnormal findings of vital signs, clinical examination, and laboratory test. Upon suspicion of AL, the radiologic tools were secured selectively for these patients.
- AL is a defect at the site of the anastomosis with subsequent communication of the intra-luminal compartment with the extraluminal one [8]. It was suggested and determined at the first record of an aberrant vital sign, laboratory finding, or upon review of radiologic tools. The postoperative complication is any deviation from the expected normal post-operative course [9]*.*

### Data collection and statistical analysis

Data acquisition was secured after reviewing the medical records of cases and all perioperative variables for each case were collected in the datasheet and were analyzed accordingly. Data analysis and interpretation were done by program SPSS v-26 (IBM, Armonk, NY). Continuous data were presented as mean and standard deviation or as median and range when appropriate, while Categorical data were presented as numbers and percentages. A Pearson’s chi-square test and Fisher’s exact test were used to compare categorical variables. Student’s t-test, the Manne Whitney U test, and one-way ANOVA were used to compare quantitative variables.

The potential relative risks for postoperative variables and Predictors of AL were assessed by univariate (using odds ratio [OR]) and multivariate (using risk ratio [RR]) analyses with 2-tailed 95% confidence interval (CI). The risk factors that were significant at a level of 10% (p < 0.10) in the univariate analysis were used to form initial multivariate model, in which the factors whose level of significance was not below 5% (p < 0.05) were sequentially excluded.

## Results

Participants and test results; the preoperative demographic data of the cases within the two groups are recorded in Table 1. The gender distribution, median age, BMI and duration of preoperative preparation did not reveal any significant difference between the two groups. The associated comorbidities were more common in the cases with AL with a statistical significance (p = 0.039). The values of pre-operative routine laboratory investigations and tumor markers are shown in Table 1 with no significant difference apart from serum albumin that was significantly lower in the leakage group vs the no-leakage one (2.9 vs 3.9).

**Table 1:** Preoperative demographic and laboratory data of the study cases

Variables	No leakage (n = 288)	Leakage (n = 27)	P value
Age (years) Mean (range)	55.5 (15–76)	54 (18–75)	0.362
Female n (%)	173 (60.1%)	13 (48.1%)	0.534
BMI(kg/m^2^)	27.66 (17.36–62.4)	28 (18.2–61.4)	0.164
Smoking n (%)	19 (6.6%)	8 (29.6%)	0.434
Hypertension n (%)	72 (25%)	10 (37%)	0.039*
DM n (%)	58 (20.1%)	12 (44.4%)	
Ischemic heart disease n (%)	24 (8.3%)	5 (18.5%)	
Haemoglobin (g/dl)	11.5 (8–16.2)	11.2 (7.5–15.6)	0.635
Creatinine (mg/dl)	0.74 (0.4–1.4)	0.69 (0.51–1.3)	0.207
Albumin (gm/dl)	3.9 (2.3–4.5)	2.9 (2.05–3.8)	0.015*
CEA (ng/ml)	2.6 (0.2–145)	3.8 (0.2–152)	0.096

*significant p value < 0.05

The analysis of the operative data is shown in Table 2. The percentage of cases with an emergency type of operation was significantly higher in the leakage group (66.7% vs 42.01%), as well as that of perforation (14.8% vs 3.2%). The other data did not reveal any significant difference between the two groups.

**Table 2:** Perioperative data of the study cases

Variables n (%)	No leakage (n = 288)	Leakage (n = 27)	P value
*Time setting of surgery*			
Elective	167 (57.99%)	9 (33.3%)	
Emergency	121 (42.01%)	18 (66.7%)	0.001*
*Indication of surgery*			0.218
Subacute IO	24 (8.3%)	3 (11.1%)	
Acute abdomen	73 (25.3%)	2 (7.4%)	
Intraabd. collection	9 (3.2%)	4 (14.8%)	
CRC	110 (38.2%)	10 (37%)	
Ileostomy closure	24 (8.3%)	1 (3.7%)	
Colostomy closure	48 (16.6%)	7 (18.5%)	
*Causes of perforation*	9 (3.2%)	4 (14.8%)	0.019*
Adenocarcinoma	3	1	
Diverticulosis	2	1	
Non-sp. inflammation	3	2	
Ischemic enteritis	1	0	
*Type of surgery*			
Transverse colectomy	9 (3.2%)	1 (3.7%)	
Lt hemicolectomy	34 (11.9%)	4 (14.8%)	0.136
Ant resection	53 (18.4%)	6 (2228%)	
Colostomy closure	48 (16.6%)	7 (18.5%)	
Small bowel resection	120 (41.7%)	10 (37.03%)	
With anastomosis			
Ileostomy closure	24 (8.3%)	1 (3.7%)	

*significant p value < 0.05

The results of the postoperative histopathological examination were shown in Table 3 which showed no significant statistical difference in the studied groups. The operative time was longer in the leakage group than the other one. The postoperative data of the cases is shown in Table 3. The percentage of cases with covering stoma was significantly higher in the no-leakage group (24.3% vs 11.1%). The median timing of the start of oral feeding and hospital stay were significantly longer in the leakage group (p = 0.038, p = 0.009). No significant differences were noted between the two groups as regards the postoperative morbidities.

**Table 3:** Perioperative variables of the outcome of the study groups

Variables n (%)	No leakage (n = 288)	Leakage (n = 27)	P value
Covering stoma	70 (% 24.3)	3(11.1%)	0.005*
*The technique*			
Stapler	38 (13.3%)	4 (14.8%)	
Hand sewn	250 (86.7%)	23 (85.2%)	0.518
Operative time (h) Mean (range)	3 (1–5)	4 (2–6)	0.065
Blood transfusion	36 (12.5%)	3 (11.1%)	0.164
*Pathology*			
Adenocarcinoma	110 (38.2%)	10 (37%)	
Diverticulosis	6 (2.1%)	1 (3.7%)	0.248
Non-specific inflammation	115 (39.9%)	11 (40.7%)	
MVO	36 (12.5%)	3 (11.1%)	
Ischemic enteritis	21 (7.3%)	2 (7.4%)	
*Oral feeding (day)* Mean (range)	4 (0–8)	5 (1–10)	0.038*
*Morbidity*			
Wound	35 (12.2%)	3 (11.1%)	
infection Ileus	148 (51.4%)	13 (48.1%)	0.154
Wound dehiscence	4 (1.4%)	3 (11.1%)	
*Hospital stay (day)* Mean (range)	6 (5–12)	10 (7–24)	0.009*

*significant p value < 0.05

According to clinical manifestations of AL (Table 4), localized peritonitis was detected in 4 cases (14.8%), diffuse peritonitis was detected in 14 cases (51.8%), wound the discharge was detected in 4 cases (14.8%), and drain discharge was detected in 5 cases (18.5%).

**Table 4:** The data set of management of anastomotic leakage cases

Variables n (%)	Leakage group (n = 27)
*The main presentation of leakage*	
Localized peritonitis	4 (14.8%)
Diffuse peritonitis	14 (51.8%)
Wound discharge	4 (14.8%)
Drain discharge	5 (18.5%)
*Radiology*	
Ultrasonography findings	
Intra-peritoneal free fluid	22 (81.5%)
Intra-peritoneal collections	5 (18.5%)
Computerized tomography findings	
Free passage of the dye	3 (11.11%)
Peri-anastomotic free fluid or collections	4 (14.8%)
Peri-anastomotic gas bubbles	3 (11.11%)
Intra-peritoneal free fluid	10 (37.03%)
Intra-peritoneal collections	7 (25.92%)
*Management of leakage*	
Conservative	9 (33.3%)
Operative;	18 (66.7%)
Repair and loop stoma	4 (14.8%)
Exteriorization of leaking site	9 (33.3%)
Covering stoma only	5 (18.5%)

The US showed a significant diagnostic ability to detect leakage as it was able to detect all cases with leakage in the form of free fluid in 22 cases (81.5%) and collection in 5 cases (18.5%). However, the US was done at the 5th POD after clinical signs and laboratory markers suggested the presence of leakage. Based on the clinical presentation and general status of the patient, the treatment was specifically tailored. Conservative treatment of the leakage was done for 9 cases who had a minor leak without peri-anastomotic contamination and minimal derangement in vital signs while the remaining 18 cases required operations as follows; repair and loop stoma in 4 cases, exteriorization of leaking site in 9 cases, and covering stoma in 5 cases.

Estimates; the lower albumin levels, Time setting of surgery, and perforation were shown in Table 5 as significant risk factors for the presence of leakage, but after using multivariate analysis for the three parameters, the emergency operation and albumin level was the only independent risk factors that revealed the significance (p = 0.017*, p = 0.042*). Other parameters in the study failed to achieve a statistically significant predictive value.

**Table 5:** analysis of the preoperative risk factors (predictors) of AL

Variables	Univariate analysis		Multivariate analysis	
	OR (95% CI)	P value	RR (95% CI)	P value
Albumin	0.46 (0.35–1.23)	0.015*	2.37 (1.823–3.94)	0.042*
Time setting of surgery (elective/emergency)	0.47 (0.25–0.98)	0.001*	2.26 (2.93–3.72)	0.017*
Covering stoma in colonic cases	0.05 (0.02–0.46)	0.003*	1.31 (0.76–2.302)	0.129

*significant p value < 0.05

**Fig. 1:**
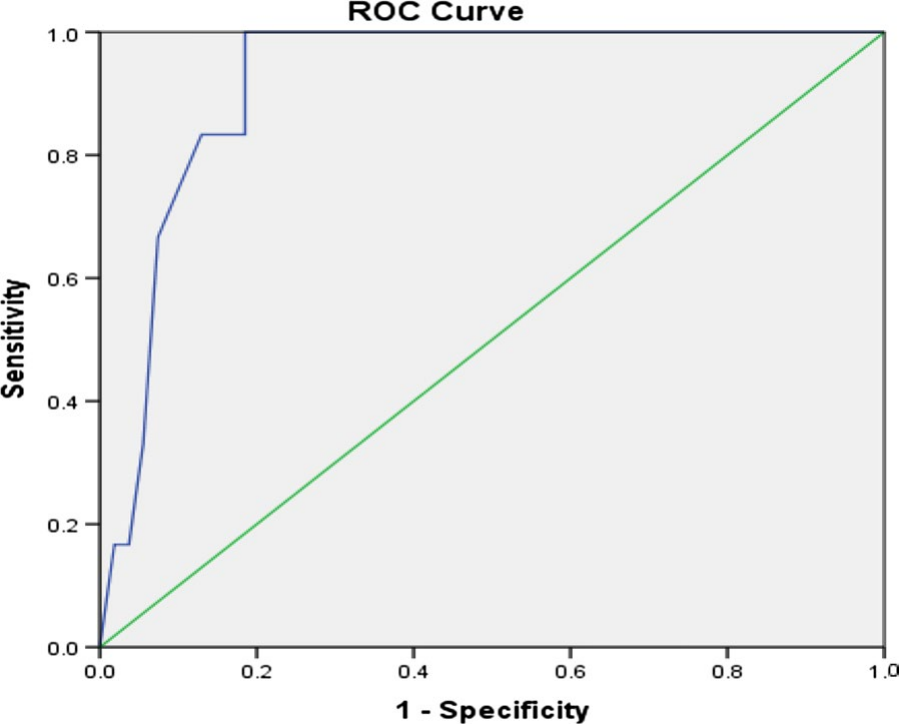
Prediction of leakage with RR (Roc curve)

**Fig. 2:**
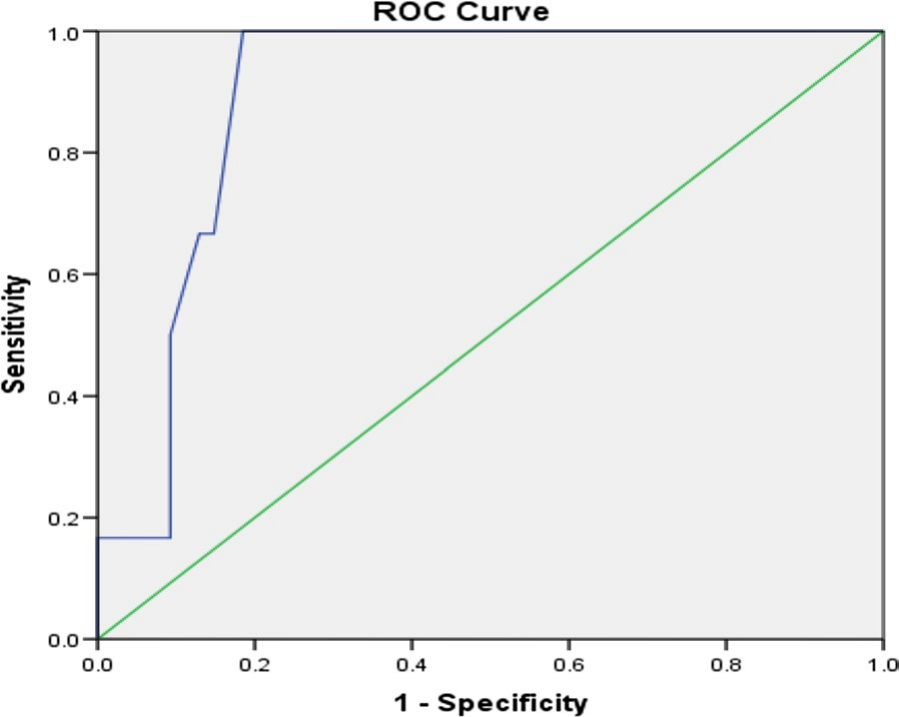
Prediction of leakage with HR (Roc curve)

**Fig. 3:**
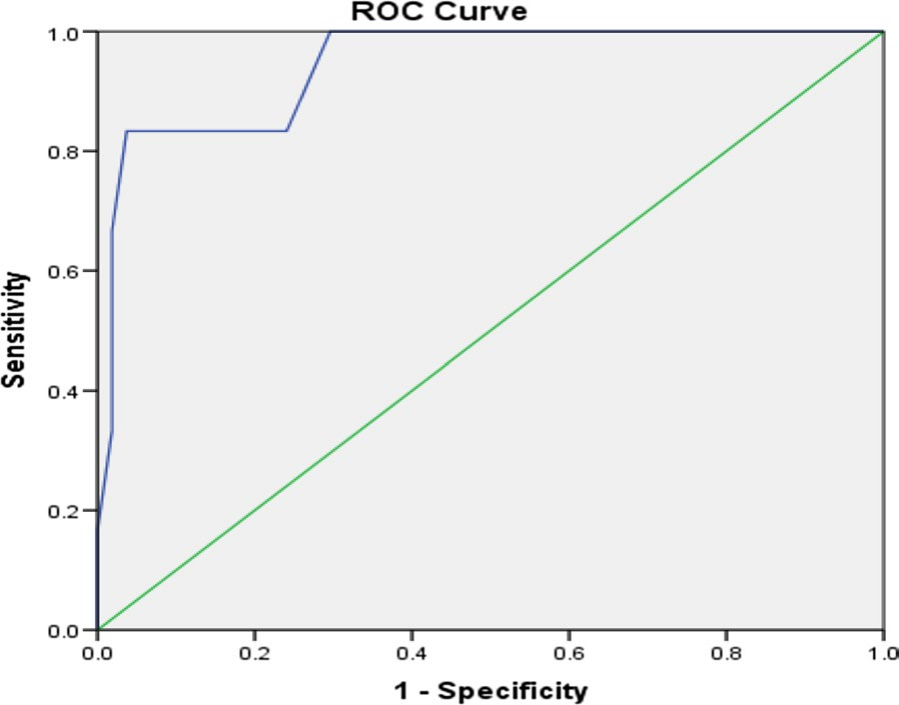
Prediction of leakage with CRP (Roc curve)

Comparison of the different postoperative laboratory and clinical parameters between the two groups is shown in Table 6. The mean HR revealed a significant difference on the 3rd and the 4th PODs (p = 0.038, p = 0.011). In the same way, the mean RR revealed a significant difference on the 3rd and the 4th PODs (p = 0.025, p < 0.001). The mean serum levels of CRP showed a statistically significant difference between the two groups beginning from the 1^st^ POD being higher in the cases with AL (p < 0.001). The analysis of different predictors of AL on the 3rd POD showed that the cut-off point in RR was 29 with 83% sensitivity and 92% specificity in prediction of AL, the cut-off point in RR was 118 with 74% sensitivity and 87% specificity in prediction of AL and the cut-off point in CRP was 184.7 with 82% sensitivity and 88% specificity in the prediction of AL and all with statistically significant value. These data are shown in Table 6 and Figs. 1, 2, 3.

**Table 6:** Analysis of the postoperative risk factors (early detection) of AL

mean ± SD	Non_leakage group	Leakage group	P value
*HR (B/min)*			
1st POD	96.04 ± 15.74	95.73 ± 13.56	p = 0.274
2nd POD	95.52 ± 10.38	97.42 ± 15.63	p = 0.141
3rd POD	93.71 ± 11.17	103.63 ± 7.94	p = 0.038*
4th POD	90.26 ± 6.86	105.91 ± 8.12	p = 0.011*
*RR (cycle /min)*			
1st POD	20.73 ± 1.86	21.37 ± 1.56	p = 0.483
2nd POD	19.62 ± 0.38	21.87 ± 1.63	p = 0.137
3rd POD	19.02 ± 0.25	23.64 ± 1.85	p = 0.025*
4th POD	18.38 ± 0.47	26.38 ± 0.95	p < 0.001**
*CRP (mg/L)*			
1st POD	35.63 ± 9.86	85.97 ± 21.56	p < 0.001**
2nd POD	81.97 ± 19.38	120.46 ± 31.02	p < 0.001**
3rd POD	107.02 ± 27.25	200.73 ± 44.2	p < 0.001**
4th POD	104.36 ± 28.47	205.27 ± 49.87	p < 0.001**

	AUC	Cut off point	P value	Sensitivity (%)	Specificity (%)	Accuracy (%)
RR	0.921	29	0.001*	83	92	89
HR	0.895	118	0.002*	74	87	85
CRP	0.872	184.7	0.009*	82	88	85

*significant p value < 0.05; **highly significant p value < 0.001

## Discussion

Despite the continual improvement in surgical techniques, AL remains one of the most devastating consequences that can occur from bowel surgery. It is associated with rising reoperation rates, length of hospital stay, morbidity, and mortality*.* The incidence of AL after colorectal resections is between 1 and 12% overall and up to 10 to 14% in low colorectal ones [10–12]*.*

The rates of morbidity and mortality significantly increase after AL, with mortality reported between 12 and 27%. Although AL has increasingly been an outcome of interest, it remains difficult to predict the risk of an individual patient. The prediction of AL can introduce a vital role in determining postoperative outcomes and can facilitate decision making in elderly patients undergoing intestinal surgeries [13–15]*.*

The objective of this research was to detect the risk factors for AL as well as its early predictors after bowel resection. So, this study included 315 cases who underwent surgery for small intestinal or colorectal disease from January 2016 till January 2019. Among these cases, 27 cases (8.57%) developed AL after the surgery. Similar results were reported by a previous study [16]*.*

In this study, the median age, gender distribution, BMI, and duration of preoperative preparation did not reveal any significant difference. The median age of the cases in the leakage group was 54 years with a range between 18 and 75 years. There were 14 males (51.8% of cases) and 13 females (48.1% of the cases) in the cases with leakage. Similar results were reported by Kream and his colleagues [17]*.*

In our study, age was not a significant predictor for AL. This is in contrast to another study included a series of 1391 cases undergoing rectal operations reported that age above 60 years is still an independent parameter for AL  [18]*.*

The associated comorbidities were significantly more common in the group of AL (p = 0.039). DM was the most common associated comorbidity and was detected in 37% and 20.1% in the cases with leakage and non-leakage group respectively, followed by HTN that was detected in 37% and 25%, respectively. Two pieces of research observed DM as an independent risk factor for AL  [19, 20]*. *Other results of literature, do not find diabetes to be a significant factor for AL [21]*.* In our study, smoking was not a significant predictor for AL. this is in contrast with several studies that have found that current smoking status is an independent predictor  [22, 23]*.*

In our study, increased BMI was not a significant predictor for AL. This is in contrast with several types of research  [24, 25]. In our study, the duration of bowel preparation was not associated with a decrease in the risk of AL. This is in agreement with several trials that have reported omitting bowel preparation does not increase the risk of AL [26, 27].

In a recent study, patient-related factors (male sex, higher ASA score, COPD, previously infected wound, steroid use, diabetes mellitus, and recent weight loss) and surgery-related factors (open technique or prolonged operation) were found to be independent predictors of AL [15] which were in accordance with the literature  [17].

In the current study, The lower albumin levels, the emergent time setting of surgery and the perforation presence was shown (Table 5) as significant risk factors for the presence of leakage, but after using multivariate analysis for the three parameters, the emergency operation, and albumin level were the only independent risk factors that revealed significance (p = 0.017*, RR 2.26 (2.93–3.72); p = 0.042*, RR 2.37(1.823–3.94)). This is in accordance with another study where the emergent setting of surgery is an independent predictor for AL (RR 4⋅6, 95% C.I. 1⋅9 to 9⋅8) [3]. Other parameters in the study failed to achieve a statistically significant predictive value.

In the present study, the type of anastomotic technique was not a predictor for the occurrence of AL. A significantly increased rate of radiological leaks in the sutured group was reported by a randomized control trial (RCT) comparing stapled with sutured anastomosis [28]*.*

In our study, the type of covering stoma was a dependent predictor for AL, but with multi regression model analysis, it was not an independent predictor for AL. The same observation was reported with a recent RCT as the leak rate was 5⋅5% in the defunctioned group compared with 37⋅5% in the one with no stoma [29]*.*

In this study, the nature of the disease whether benign or malignant did not affect the occurrence of AL. This was in accordance with Park et al. [30]where the nature and the stage of the disease did not affect the incidence of AL.

In our study, the operative time and the intraoperative blood loss were not shown to be predictors for the occurrence of AL. Our results came in agreement with the literature that reported an increased length of hospital stay (13 vs 5 d; *p* < 0.001), higher readmission (43.5% vs 8.3%; *p* < 0.001), and reoperation rates (*p* < 0.001) in patients with colectomy who had AL compared with those who did not  [31]*.*

In this study, According to postoperative pathology, types of pathological disease failed to achieve any significant difference between the leakage and the non-leakage group even MVO. Our protocol was to take a wide safety margin to avoid impending ischemic segments as the normal healing process of the anastomosis to take place, it must have ample tissue perfusion  [32]*.*

The current study evaluated the effect of AL on postoperative outcomes. The same results were noted in Patients in whom AL developed had a longer hospital stay, more postoperative complications, a longer time for the return of bowel functions, and higher readmission rates [15].

According to the main presentation of AL, localized peritonitis was detected in 4 cases (14.8%), diffuse peritonitis was detected in 14 cases (51.8%), wound discharge was detected in 4 cases (14.8%) and drain discharge was detected in 5 cases (18.5%). This was in agreement with the results from the literature reported that general peritonitis is one of the most severe outcome for AL [33].

Different clinical presentations can be manifested with AL ranging from abdominal discomfort, and fever, up to severe sepsis. Altered sensorium with pulmonary abnormalities is commonly presented early in AL [34].

In this study, US and CT showed a significant diagnostic ability to detect leakage as both of them were able to detect all cases with leakage in the form of free fluid and collection. Radiologic findings were frequently demanding proper correlation with the clinical presentation. When the radiologic studies appear to be negative, a high index of suspicion should be maintained. As on the one hand, one study found that the sensitivity for contrast-enhanced CT scan is in the range of 50% in the setting of a leak [35]. The overlapping of radiologic findings is commonly encountered in patients with or without a leak. Only loculated fluid with air was reported more frequently in patients with AL [36].

In our study, the mean postoperative HR showed a significant difference between the two groups on the 3rd and 4th PODs (p = 0.038, p = 0.011). In the same way, the mean RR revealed a significant difference between the two groups on the 3rd and 4th PODs (p = 0.025, p < 0.001). On the contrary, the number of WBCs and body temperature did not show a significant difference in the period of postoperative follow-up for 4 days. Our findings were consistent with previous research which found that fever and leukocytosis were not commonly present, and when present manifested in the late PODs  [34].

In our study, Conservative treatment of the leakage was done for 9 cases while the remaining 18 cases required operations as follows; repair and loop stoma in 4 cases, exteriorization of leaking site in 9 cases, and covering the stoma in 5 cases. This was in agreement with Nikolian et al. [37] who found that most of the cases diagnosed with major AL underwent re-operative interventions. Also, the reoperation rate for AL in our study is consistent with previously published research [38]*.*

Also, in this study, the mean serum levels of CRP revealed a statistically significant difference between the cases with leakage and no-leakage beginning from the 1st POD being higher in the cases with AL (p < 0.001). Also, in our study, all postoperative HR, RR, and CRP were significant predictors for the occurrence of AL with a sensitivity of 83%, 74%, and 82% respectively.

The modified DULK score of AL included; RR ˃20 breaths/min, clinical deterioration, abdominal pain (other than wound pain), and CRP level ˃ 250 mg/l. However, CRP is not sensitive to the detection of AL because it may be increased with any other infection [7].

In our study, the analysis of different predictors of AL on the 3rd day showed that the cut-off point in RR was 29 with 83% sensitivity and 92% specificity in prediction of leakage, the cut-off point in RR was 118 with 74% sensitivity and 87% specificity in prediction of leakage and the cut-off point in CRP was 184.7 with 82% sensitivity and 88% specificity in prediction of leakage and all have statistically significant values.

The current study had some limitations, as it was only a two-center study, and the sample size may be considered relatively small, which restricts the power of conclusions. Also, the study included a heterogeneous group of patients of small intestinal and colorectal surgery, since anastomoses in these locations lead to different numbers of leakage rates with different pathology. Moreover, they have different pathophysiological pathways underlying AL with different aetiological factors. Therefore, wide further improvements are needed in other future studies. Large numbers of homogenous series are suggested to confirm these results and proceed for establishing a new score for prediction and early detection of AL.

## Conclusion

The preoperative serum albumin level and emergency operations are independent risk factors for anastomotic leakage. Moreover, leakage should be suspected and predicted in cases with a high respiratory rate, heart rate, and CRP levels.

## Data Availability

The datasets used and/or analysed during the current study are available from the corresponding author on reasonable request.

## Abbreviations

AL: Anastomotic leaks
POD: Postoperative day
ASA: American Society of Anesthesia
BMI: Body mass index
COPD: Chronic obstructive pulmonary disease
MIH: Mansoura University Hospitals
MIH: Mansoura International Hospital
RCT: Randomized control trial
DM: Diabetes mellitus
CRP: C-reactive protein
LDH: Lactic dehydrogenase
ABG: Arterial blood gases
RR: Respiratory rate
HR: Heart rate
IO: Intestinal obstruction
CRC: Colorectal carcinoma
MVO: Mesenteric vascular occlusion
OR: Odds ratio
RR: Risk ratio
CI: Confidence interval
ROC: Receiver operating characteristic
AUC: Area under curve
